# Albiglutide, a Long Lasting Glucagon-Like Peptide-1 Analog, Protects the Rat Heart against Ischemia/Reperfusion Injury: Evidence for Improving Cardiac Metabolic Efficiency

**DOI:** 10.1371/journal.pone.0023570

**Published:** 2011-08-26

**Authors:** Weike Bao, Karpagam Aravindhan, Hasan Alsaid, Thimmaiah Chendrimada, Matthew Szapacs, David R. Citerone, Mark R. Harpel, Robert N. Willette, John J. Lepore, Beat M. Jucker

**Affiliations:** 1 Heart Failure Discovery Performance Unit, Metabolic Pathways and Cardiovascular Therapy Area Unit, GlaxoSmithKline, King of Prussia, Pennsylvania, United States of America; 2 Clinical Imaging Center, GlaxoSmithKline, King of Prussia, Pennsylvania, United States of America; 3 Platform Technology and Science, GlaxoSmithKline, King of Prussia, Pennsylvania, United States of America; University of Padova, Italy

## Abstract

**Background:**

The cardioprotective effects of glucagon-like peptide-1 (GLP-1) and analogs have been previously reported. We tested the hypothesis that albiglutide, a novel long half-life analog of GLP-1, may protect the heart against I/R injury by increasing carbohydrate utilization and improving cardiac energetic efficiency.

**Methods/Principal Findings:**

Sprague-Dawley rats were treated with albiglutide and subjected to 30 min myocardial ischemia followed by 24 h reperfusion. Left ventricle infarct size, hemodynamics, function and energetics were determined. In addition, cardiac glucose disposal, carbohydrate metabolism and metabolic gene expression were assessed. Albiglutide significantly reduced infarct size and concomitantly improved post-ischemic hemodynamics, cardiac function and energetic parameters. Albiglutide markedly increased both *in vivo* and *ex vivo* cardiac glucose uptake while reducing lactate efflux. Analysis of metabolic substrate utilization directly in the heart showed that albiglutide increased the relative carbohydrate versus fat oxidation which in part was due to an increase in both glucose and lactate oxidation. Metabolic gene expression analysis indicated upregulation of key glucose metabolism genes in the non-ischemic myocardium by albiglutide.

**Conclusion/Significance:**

Albiglutide reduced myocardial infarct size and improved cardiac function and energetics following myocardial I/R injury. The observed benefits were associated with enhanced myocardial glucose uptake and a shift toward a more energetically favorable substrate metabolism by increasing both glucose and lactate oxidation. These findings suggest that albiglutide may have direct therapeutic potential for improving cardiac energetics and function.

## Introduction

Glucagon-like peptide-1 (GLP-1) is an incretin hormone secreted by intestinal L-cells in response to nutrient ingestion [1]. GLP-1 occurs in a number of biologically active isoforms including full length GLP-1 (7–36) amide, a glycine-extended isoform of GLP-1 (7–37), and the N terminus cleaved GLP-1 (9–36). GLP-1 regulates glucose homeostasis by stimulating insulin secretion, inhibiting glucagon secretion, delaying gastric emptying and promoting satiety [2]. Although the major physiological function of GLP-1 is associated with glycemic control, increasing evidence indicates that GLP-1 may also play an important role in cardiovascular physiology [3]. It has been reported that GLP-1 receptors are expressed in both heart and coronary vasculature and activation of GLP-1 receptors by agonists results in a wide range of cardiovascular effects such as cardioprotection against myocardial ischemia-reperfusion injury both *ex vivo* [4]–[7] and *in vivo* [5].

GLP-1 has a very short half-life of about 2 min following exogenous administration as it is rapidly cleaved and inactivated in plasma by the protease dipeptidyl peptidase-IV (DPP-IV). This short half-life limits its use as a therapeutic agent. However, GLP-1 analogs that mimic the effect of GLP-1 but are resistant to DPP-IV have been explored for the treatment of type 2 diabetes [2], [8]. Despite consistent effects of GLP-1 analogs on glycemic control observed in preclinical and clinic studies, the effect on myocardial I/R injury remains controversial [9]–[11]. It is of clinical relevance to further validate the cardioprotection and explore its novel protective mechanism of GLP-1 analogs as these novel GLP-1 analog agents are being developed for a diabetic patient population whom are also at high risk for cardiovascular disease.

Recent studies indicate that increased cardiac glucose uptake is beneficial in protecting the heart against ischemic injury [12]. It is well known that GLP-1 receptor activation leads to insulinotropic and insulinomimetic effects in various tissues including the heart [2], [13]–[15]. In addition, a few studies have been performed *in situ* [13], [14] and *ex vivo* [6], [7] to assess the beneficial effects of GLP-1 on glucose uptake by the heart. However, the intermediary carbohydrate metabolism and overall effect on cardiac energetics following GLP-1 receptor activation have not been previously described. Albiglutide consists of two copies of a 30 amino acid sequence of human glucagon-like peptide 1 (GLP-1, fragment 7–36) that is DPP-IV resistant (alanine to glycine conversion at amino acid 8) fused with human albumin to provide a long half-life of ∼6–8 days [17]. Albiglutide is currently being investigated in a phase III clinical trial for its potential to treat type II diabetes [18]. In the present study, we hypothesized that albiglutide, a novel long half-life GLP-1 analog, may exert cardioprotective effects in a rodent model of myocardial I/R injury by shifting cardiac energy metabolism toward more efficient carbohydrate utilization, thereby improving cardiac energetics and increasing cardiac function.

## Materials and Methods

### Myocardial ischemia/reperfusion injury, hemodynamic measurement and postmortem analysis in Sprague-Dawley rats

Male Sprague-Dawley rats purchased from Charles-River laboratory (Wilmington, DE), were used for the study. All animal studies were performed in compliance with the Guide for the Care and Use of Laboratory Animals as published by the US National Institutes of Health and were approved by the Institutional Animal Care and Use Committee of GlaxoSmithKline. Rats were treated with vehicle or albiglutide at 1, 3 or 10 mg/kg/day (n = 8–10 per group) by daily subcutaneous injection for 3 days. The last dose was administered 2 h before ischemia. Rats underwent 30 min myocardial ischemia via left anterior descending artery occlusion followed by 24 h reperfusion as previously described [19], [20]. Rat hemodynamics and myocardial infarct size were determined as previously described [19], [20]. All metabolic mechanism studies were conducted with 3 days of dosing at 10 mg/kg/day unless otherwise indicated.

### Cardiac glucose uptake measurement *in vivo* and *ex vivo*

Cardiac glucose disposal *in vivo* was conducted as previously described in conscious rats [21], [22]. Briefly, rats were dosed with albiglutide as described above prior to administration of 2-[^3^H] deoxyglucose (2-[^3^H] DG) intravenously (n = 14–15 per group). Glucose disposal flux was determined using the average plasma glucose concentration, terminal heart tissue radioactivity, and plasma glucose radioactivity AUC (area under the curve) [21], [22]. To further examine the direct role of albiglutide on cardiac glucose disposal, dosing was performed as described above prior to harvesting the heart and performing the Langendorff perfused heart preparation as previously described [6], [23]. The glucose flux across the heart was determined as (concentration of glucose (initial)−concentration (final))×(perfusion volume/heart wet weight (g)/time (h)); Lactate flux was determined as (concentration of lactate (final)−concentration (initial))×(perfusion volume/heart wet weight (g)/time (h)) [6].

### Cardiac metabolic flux assessment

Two separate experiments were performed to examine cardiac intermediary metabolism: 1) relative carbohydrate vs. free fatty acid oxidation was assessed following an *in vivo* glucose clamp experiment, and 2) lactate flux and oxidation were assessed in the Langendorff perfused heart.

For the glucose clamp experiment, rats (n = 5 per group) were treated with albiglutide as described above prior to being subjected to a euinsulinemic-hyperglycemic clamp (continuous [1-^13^C] glucose (8 mg/kg/min)/somatostatin (1.5 µg/min) infusion via jugular vein) under awake conditions for 120 min. The POCE (Proton Observe Carbon Enhanced) ^1^H magnetic resonance spectroscopy (MRS) measurements of metabolite ^13^C enrichments in tissue extracts were performed using a 9.4T spectrometer as previously reported [19], [24]–[26]. Relative cardiac carbohydrate (including glucose, glycogen, pyruvate, and lactate) and free fatty acid (FFA)/ketone oxidation in terms of relative substrate contribution to acetyl-CoA oxidation was assessed from the metabolite pool enrichments as follows: the relative carbohydrate oxidation rate was calculated as: (4-^13^C glutamate enrichment)/(3-^13^C alanine enrichment), and the relative fat oxidation is calculated as: 1-(4-^13^C glutamate enrichment)/(3-^13^C alanine enrichment).

For assessing lactate oxidation and flux, a perfused heart experiment was performed in which 3-^13^C lactate was used as a precursor for lactate oxidation measurements. Rats were treated with albiglutide as described above prior to harvesting hearts. Albiglutide (0.12 µM) or vehicle (n = 10/group) was added to a modified KH solution containing 3% BSA (essentially fatty acid free), 1 mM 3-^13^C sodium lactate, 0.1 mM sodium pyruvate, 0.4 mM sodium palmitate, and 11 mM glucose. The heart was perfused for 60 minutes during which influent and effluent samples were collected and lactate concentration was analyzed using an Olympus AU640 analyzer. In addition, influent and effluent 3-^13^C lactate enrichments and heart tissue 3-^13^C lactate, 3-^13^C alanine, and 4-^13^C glutamate enrichments were measured by MRS and relative lactate oxidation as substrate contribution to acetyl-CoA was calculated as: (4-^13^C glutamate enrichment)/(3-^13^C alanine enrichment).

### *In vivo* cardiac energetic assessment

To examine the effect of albiglutide on the cardiac energetic profile (e.g. ATP, PCr, ATP/PCr) following cardiac I/R injury, animals were treated with albiglutide or vehicle and then subjected to 30 min ischemia followed by 24 hrs reperfusion as described above. Cardiac MRI/MRS was performed at 24 hrs post-reperfusion using a double tune (^1^H, ^31^P) concentric surface radio frequency (RF) coil (m2m Imaging, Newark, NJ) setup on a 9.4T/30 cm Bruker Biospec system (Billerica, MA). A cine loop was generated for each imaging slice through the ventricles with a sufficient number of delays to cover the cardiac cycle. The imaging parameters were as follows: matrix dimensions, 128×128; TR/TE, 7/1.5 ms; slice thickness, 2.0 mm; FOV, 5.0 cm; number of repetition 250; cine loop, 10 images. Left ventricle (LV) functional parameters were analyzed (Analyze AVW software, AnalyzeDirect, Lenexa, KS). ^31^P MRS was performed immediately following the imaging using the same RF hardware and spatially localized spectroscopy of the heart was performed using a 3-D Image Selected *In Vivo* Spectroscopy (ISIS) sequence with outer volume suppression (TR = 4 s, NS = 512, SW = 10 kHz, 1 k data). The spectroscopic voxel of interest size was 15.8×11×17 mm and positioned to cover the LV in an oblique plane in all three orthogonal directions. ^31^P NMR spectra were processed using an exponential filter and baseline flattening. The phosphocreatine (PCr) peak, was set to 0 ppm, and the inorganic phosphate (P_i_) peaks at 4.9–5.3 ppm (including both intracellular and extracellular P_i_) and the β-adenosine triphosphate (ATP) peak at −16 ppm were integrated in order to calculate the PCr/P_i_ and PCr/ATP ratios respectively (Nuts NMR processing software, Acorn NMR Inc., Fremont, CA). The intracellular pH was calculated using the chemical shift difference between intracellular P_i_ and PCr as previously described [27]. Absolute concentrations of ATP and PCr were extrapolated using an external concentration standard.

### Biochemical analysis, clinical chemistry and albiglutide pharmacokinetics

Tissue cAMP was measured using an EIA kit from Cayman Chemical (Cat#581001). Cardiac glycogen was measured as previously described [28]. Plasma insulin was measured using MDS rat insulin Kit (K152BZC-2). Glucose and lactate were measured using an Olympus AU 640 analyzer (Olympus America Inc., Melville, NY). Rat plasma samples were analyzed for albiglutide using an analytical method based on sample dilution followed by immunoassay analysis.

### Cardiac metabolic gene expression analysis

Quantitative TaqMan reverse-transcription (RT) polymerase chain reaction (PCR) analysis was performed on total RNA that was extracted from LV in a subgroup of SD rats that were treated with vehicle or albiglutide as described above (n = 8 biological replicates and n = 2 technical replicates per group). Twenty four key metabolic, mitochondrial, and stress associated genes were assessed in normal LV as well as ischemic and non-ischemic LV from I/R experiment. These genes consisted of: ACSL1, CPT1B, GAPDH, IGF1R, SLC2A4, HK2, GYS1, ESSRA, HIF1A, PPARGC1A, PDHA1, ALDOC, GSK3B, AKT1, SLC2A1, PYGL, PRKAA2, PPARG, HK1, PDK1, UCP2, ACTB, LDHA, UCP3. Gene expression data was normalized using the Omics Studio® software. One-way ANAOVA and PCA were performed thereafter.

### Statistical analysis

Data are presented as mean ± SEM. Differences between groups were compared by paired and unpaired Student's t tests or by a one-way ANOVA followed by a Bonferroni test for multiple comparisons. A p value of <0.05 was considered statistically significant.

## Results

### Body weight, food consumption, and plasma levels of insulin, glucose, lactate and albiglutide

Albiglutide significantly reduced body weight and food consumption in a dose-dependent manner which is consistent with the known pharmacology of GLP-1 mimetics (Table 1). Albiglutide also increased plasma insulin and concomitantly decreased plasma glucose compared to vehicle at all doses. Plasma lactate was elevated at the higher doses (3 and 10 mg/kg) of albiglutide. The terminal plasma concentrations of albiglutide were 7.9±1.7 nM, 17±1.8 nM and 118±18.8 nM at the 1, 3 and 10 mg/kg doses, respectively (Table 1).

**Table 1:** Clinical chemistry profile in rats following 3 days of albiglutide administration.

	Vehicle	Albiglutide (mg/kg)		
		1	3	10
Body weight (g)	279±6	278±4	293±7	290±8
Body weight change (g/day)	7±1	8±1	5±1	−5±1*
Insulin (pg/ml)	446±160	2546±500*	1744±491	1987±418*
Glucose (mg/dL)	214±5	166±4*	170±7*	157±7*
Lactate (mg/dL)	13±1	14±1.7	17±1.4†	21±2.1*
Terminal albiglutide concentration (nM)	—	7.9±1.7	17±1.8	118±18.8

Data are presented as mean ± SEM, n = 8–10 per group.

*p<0.01 vs. vehicle;

†p<0.05 vs.vehicle.

### Myocardial infarct size, cAMP concentration, and function following ischemia/reperfusion injury

**Figure 1:**
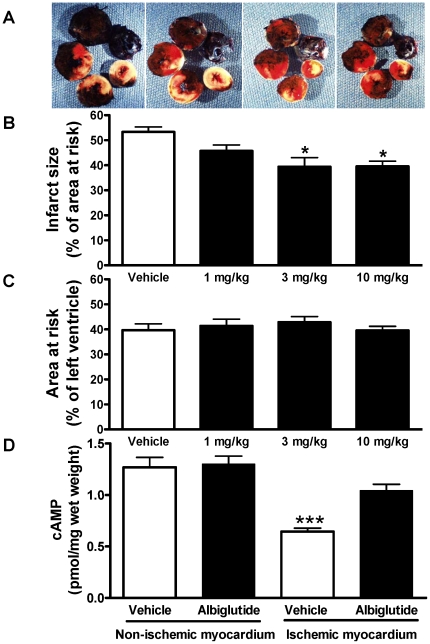
Infarct size following myocardial ischemia/reperfusion injury. Sprague-Dawley rats were subjected to 30 min LAD coronary artery occlusion followed by 24 h reperfusion. Hearts were harvested and analyzed for area at risk and infarct size. Representative photographs of heart sections from vehicle, 1, 3, and 10 mg/kg albiglutide-treated animals stained with 2,3,5-triphenyltetrazolium chloride (TTC) and Evans blue dye illustrate myocardial infarct (white), area at risk (white and red) and area not at risk (dark blue) (A). Myocardial infarct size was assessed as a percentage of area at risk in the left ventricle (B). The area at risk was assessed as a percentage of the left ventricle area is shown in (C). Cardiac cAMP was measured in both ischemic and non-ischemic areas (D). Values are presented as mean ± SEM. *p<0.01 and ^†^p<0.001 vs. vehicle.

The myocardial infarct size following I/R injury averaged 53.3±2.0% of the area at risk in the vehicle group after I/R injury. Treatment with albiglutide significantly reduced infarct size compared to vehicle: Infarct size was 45.7±2.4%, 39.4±3.7%, and 39.5±2.1% of area at risk at doses of 1, 3 and 10 mg/kg, respectively (all p<0.05, Figure 1A, B; Table 2). The area at risk was similar between vehicle- and albiglutide-treated animals (Figure 1C). There were no differences in heart rate or LV systolic pressure between vehicle- and albiglutide-treated animals (Table 2). However, the increased LV end diastolic pressure induced by I/R injury was significantly blunted with albiglutide (10 mg/kg) and the decreased contractile function was significantly improved with albiglutide treatment (3, 10 mg/kg) compared to vehicle (Table 2). cAMP levels in the ischemic and non-ischemic regions of the heart were measured in a separate group of animals. While cAMP concentrations were not elevated following albiglutide treatment (10 mg/kg) in the non-ischemic LV, cAMP levels were normalized in the ischemic region by albiglutide (Figure 1D).

**Table 2:** Infarct size, ischemic area and hemodynamics in rats following 3 days of albiglutide administration.

	Vehicle	Albiglutide (mg/kg)		
		1	3	10
Infarct size (% of area at risk)	53±2.0	45.7±2.4	39.4±3.7*	39.5±2.1*
Area at risk (% of left ventricle)	39.7±2.5	41±2.7	42.9±2.2	39.6±1.6
Heart rate (bpm)	408±14	386±9	412±8	409±12
LVSP (mmHg)	94±3	94±4	99±2	102±3
LVEDP (mmHg)	11±1	10±1	10±1	8±1*
dP/dt _max_ (mmHg/s)	5921±194	6112±434	7033±257*	7461±475†
dP/dt _min_ (mmHg/s)	4924±297	4978±395	5436±224	6254±537

Data are presented as mean ± SEM, n = 8–10 per group.

*p<0.01 vs. vehicle;

†p<0.05 vs. vehicle.

### Cardiac glucose disposal and intermediary metabolism

**Figure 2:**
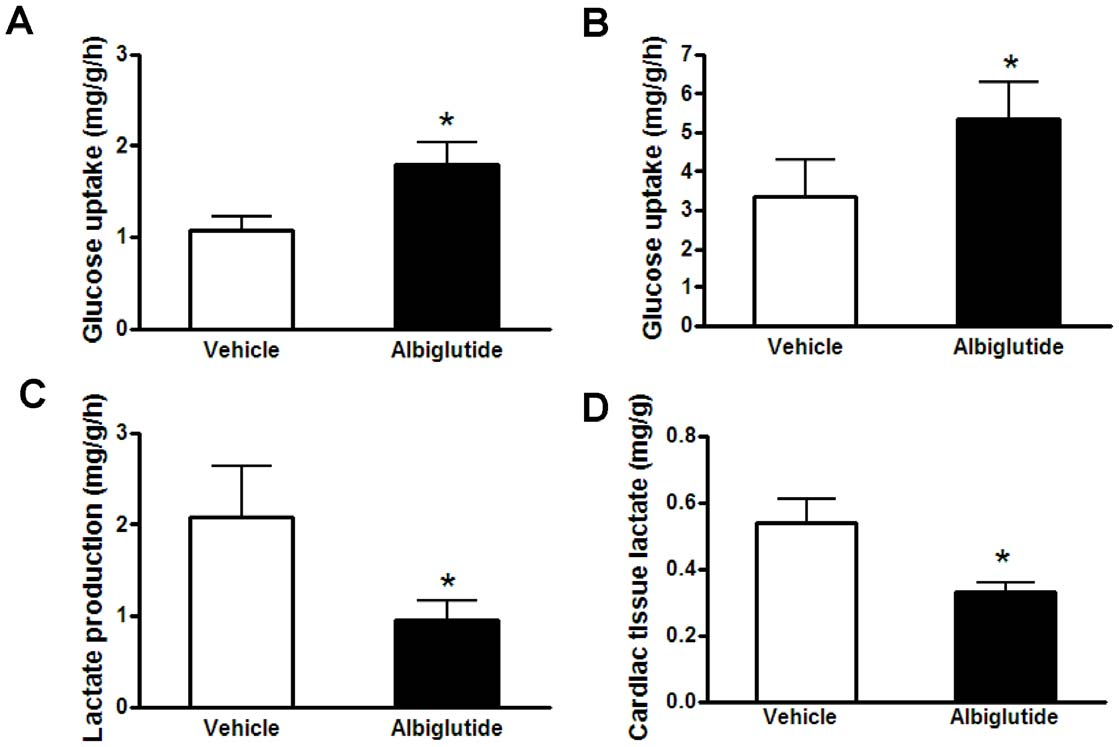
Cardiac glucose metabolism *in vivo* and *ex vivo*. Cardiac [^3^H]-2-deoxyglucose uptake was examined *in vivo* over a 30 min period (A). Additionally, cardiac glucose uptake (B), lactate production (C) and tissue lactate concentration (D) were measured directly in the Langendorff perfused hearts. Values are presented as mean ± SEM. *p<0.05 vs. vehicle.

To determine if increased myocardial glucose uptake is associated with albiglutide treatment, *in vivo*, [^3^H]-2-deoxyglucose uptake was determined. As shown Figure 2A, 2-DG uptake in the heart was significantly increased by 67% following treatment with albiglutide when compared to vehicle (p<0.05).

To further assess the direct versus indirect effects of albiglutide on cardiac glucose uptake, *ex vivo* cardiac glucose disposal and lactate flux were evaluated in the Langendorff perfused heart using the same *in vivo* dosing protocol as described above and followed by a continuous perfusion of albiglutide at 0.12 µM (equivalent to the 10 mg/kg dose concentration in the *in vivo* study). The glucose disposal was increased by 59% (p<0.05) in the albiglutide-treated hearts compared to the vehicle treated hearts (Figure 2B). Interestingly, lactate efflux was decreased by 55% in albiglutide-treated hearts compared to vehicle-treated hearts (p<0.05, Figure 2C). The cardiac tissue lactate concentration at the end of the study was also significantly lower (↓41%, p<0.05) in albiglutide versus vehicle-treated hearts (Figure 2D).

**Figure 3:**
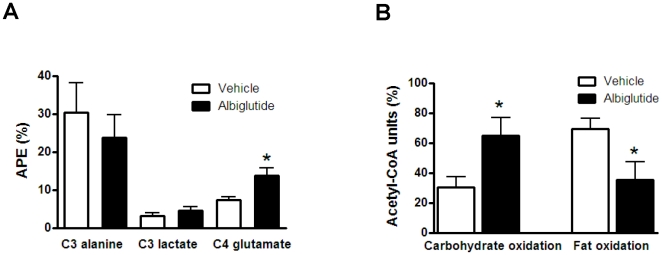
*In vivo* intermediary glucose metabolism in normal rat hearts. A euinsulinemic-hyperglycemic clamp was performed for 2 h using 1-^13^C glucose as the exogenous precursor. LV intermediary metabolite ^13^C enrichments of alanine, lactate, and glutamate are presented as atom percent excess (APE) (A). The intermediary metabolite enrichments were used to indirectly assess the relative metabolic flux through carbohydrate versus fat oxidation as a percentage of flux through acetyl-CoA (B). Values are presented as mean ± SEM. *p<0.05 vs. vehicle.

To determine the effect of albiglutide on cardiac intermediate glucose metabolism, metabolic substrate utilization was assessed using a 1-^13^C glucose euinsulinemic-hyperglycemic clamp. There were no differences in alanine and lactate enrichments between vehicle- and albiglutide-treated hearts (Figure 3A). However, enrichment of 4-^13^C glutamate, a TCA cycle intermediate, was significantly higher in the albiglutide-treated heart compared to vehicle-treated heart reflecting increased glycolytic flux into the TCA cycle (Figure 3A). Furthermore, treatment with albiglutide resulted in a significantly increased relative ratio of carbohydrate versus fat oxidation (↑112%, p<0.05 vs. vehicle) as calculated by isotopomer analysis of alanine and glutamate enrichments (Figure 3B). Cardiac glycogen levels were similar in both albiglutide and vehicle treated hearts (12.2±1.7 vs. 11.4±3.5 µmol/g, respectively). However, albiglutide did not alter the metabolic profile in the ischemic heart (data not shown).

**Figure 4:**
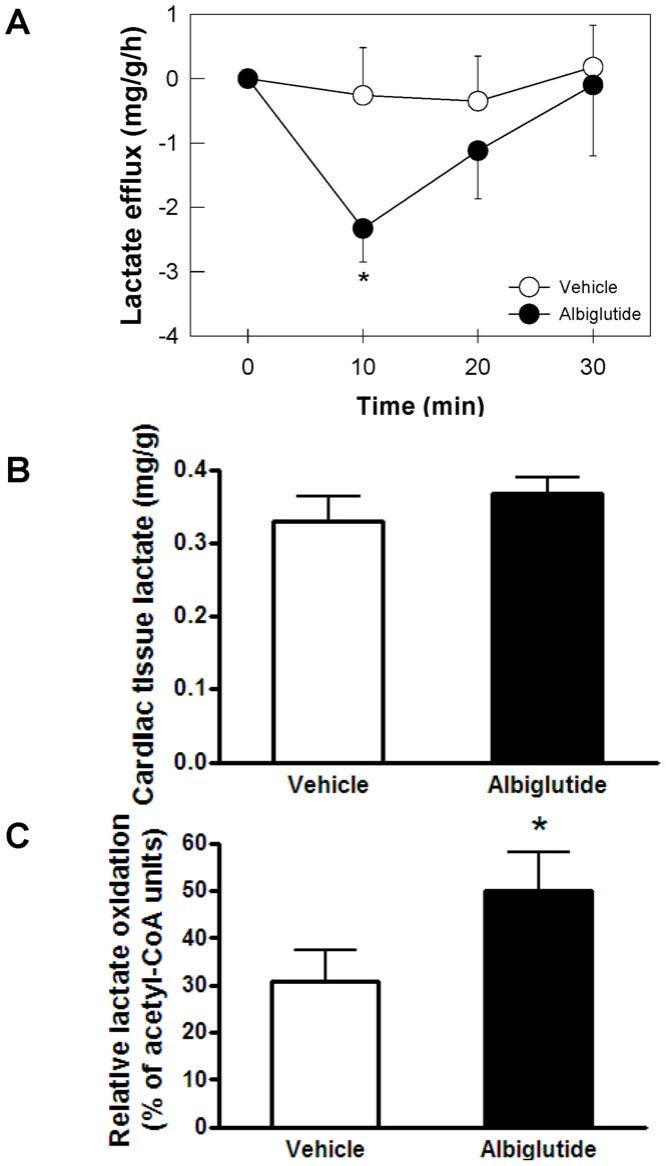
Lactate disposition *ex vivo*. Net lactate efflux across the heart during a 30 min perfusion period using a 1 mM 3-^13^C lactate precursor (A), cardiac tissue lactate concentration (B), and relative lactate oxidation as reflected by increased glutamate labeling (C). Values are presented as mean ± SEM. *p<0.05 vs. vehicle.

To further explore the effect of albiglutide treatment on cardiac lactate metabolism, 3-^13^C lactate cardiac flux and oxidation were assessed in the Langendorff perfused heart under physiological conditions of substrate availability (i.e. inclusion of glucose, lactate, pyruvate, FFA at physiological levels). Albiglutide transiently increased lactate influx into the perfused heart over 30 min (Figure 4A) (negative efflux) while having no effect on cardiac lactate concentration in this experiment (Figure 4B). Yet, albiglutide treatment resulted in an increased relative lactate oxidation compared to vehicle (↑63%, p<0.05, Figure 4C).

### Metabolic gene transcription

**Figure 5:**
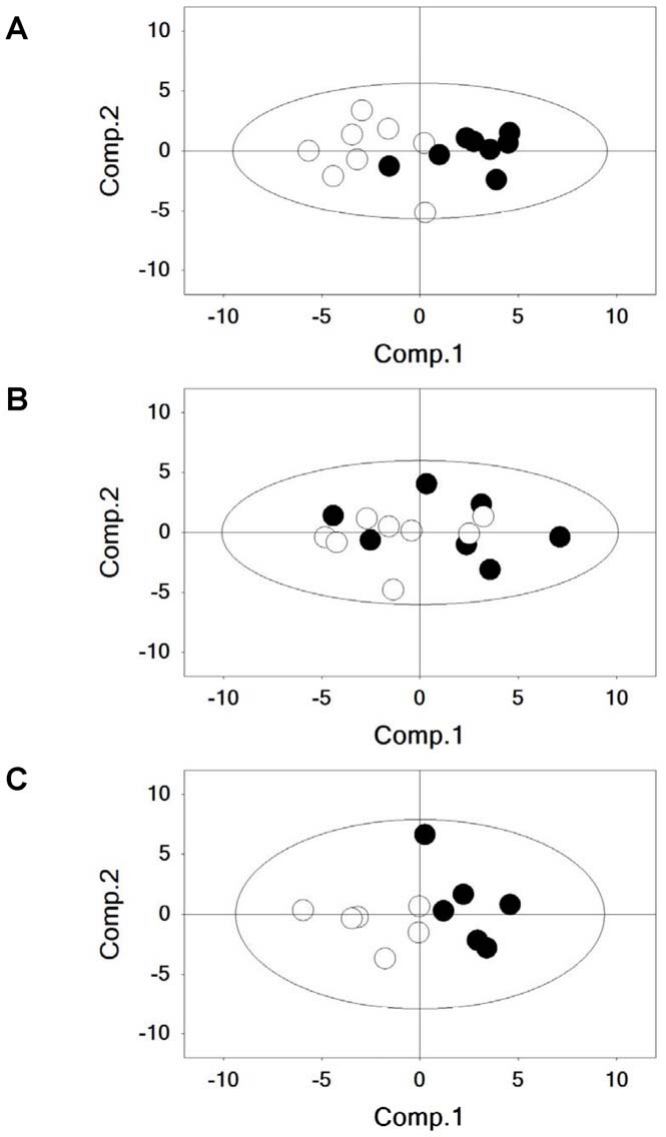
Principal Component Analysis of metabolic genes. Principal Component Analysis of the gene expression data collected from normal and ischemia injured heart samples was performed and presented for normal hearts (A) and area at risk hearts following 30 min myocardial ischemia and 24 h reperfusion (B) and area not at risk hearts from the same animals (C).

Taqman® RT-PCR analysis was performed using a refined panel of 24 genes involved in regulation of glucose and fat metabolism with both normal and I/R injured heart tissues. ACSL1, CPT1B, GAPDH, IGF1R, SLC2A4, HK2, GYS1, ESSRA, HIF1A, PPARGC1A, PDHA1, ALDOC, GSK3B, AKT1 increased significantly in normal hearts following treatment with albiglutide (Table 3). Although fewer genes (GYS1, SLC2A4) were upregulated significantly in the ischemic region of the I/R hearts, several genes associated with glycolysis and glycogenesis (ALDOC, CPT1B, IGF1R, PDHA1, GSK3B, GYS1, SLC2A1) increased significantly in the non-ischemic region of the I/R hearts following treatment with albiglutide. Principal Component Analysis (PCA) of the gene expression data collected from all three heart tissue samples suggests clustering of both normal (non-infarcted hearts) and area not at risk (infarcted hearts) tissue expression data following treatment with albiglutide, which is consistent with a definite alteration in regulation of glucose and fat metabolism genes in both normal and non-ischemic regions of the heart (Figure 5).

**Table 3:** Effect of albiglutide on metabolic gene transcriptional changes in the heart.

GENE	Gene Name	Albiglutide vs. Vehicle(fold change)	p Value
ACSL1	Acetyl CoA Synthase 1	1.42	0.0003
CPT1B	Carnitine Palmitoyltransferase 1B (muscle)	1.44	0.0004
GAPDH	Glyceraldehyde 3-Phosphate Dehydrogenase	1.24	0.0004
IGF1R	Insulin Growth Factor 1R	1.36	0.0005
SLC2A4	Glucose Transporter 4	1.34	0.0006
HK2	Hexokinase 2	1.41	0.0007
GYS1	Glycogen Synthase	1.21	0.0012
ESSRA	Estrogen Receptor, alpha isoform	1.29	0.0022
HIF1A	HIF-1 α	1.22	0.0045
PPARGC1A	PGC-1α	1.28	0.0085
PDHA1	Pyruvate Dehydrogenase	1.17	0.0116
ALDOC	Fructose 1, 6 Bisphosphate Aldolase	1.36	0.0120
GSK3B	Glycogen Synthase Kinase	1.16	0.0378
AKT1	AKT1	1.13	0.0413
SLC2A1	Glucose Transporter 1	1.24	0.0570
PYGL	Glycogen Phosphorylase	−1.33	0.1158
PRKAA2	AMPK	1.06	0.1820
PPARG	PPAR Gamma	1.12	0.1862
HK1	Hexokinase 1	1.06	0.2117
PDK1	Pyruvate Dehydrogenase Kinase	1.28	0.2247
UCP2	Uncoupling Protein 2	1.16	0.4040
ACTB	β- Actin	1.03	0.6339
LDHA	Lactate Dehydrogenase-1 (LDH-1)	1.02	0.7039
UCP3	Uncoupling Protein 3	−1.04	0.8503

Gene transcription regulation in albiglutide vs. vehicle treated hearts. Data are presented as fold change vs. vehicle, n = 8 per group.

### Cardiac energetics

**Figure 6:**
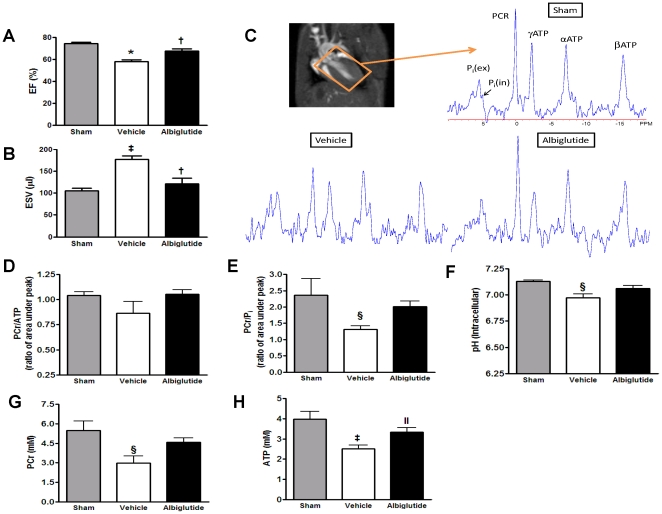
Cardiac function and high energy metabolites following myocardial ischemia/reperfusion injury. LV ejection fraction (A) and LV end systolic volume (B) were assessed by MRI immediately following the ^31^P MRS measurement at 24 h post-reperfusion. A voxel (15.8×11×17 mm) was orthogonally positioned in all 3 scout image planes of the heart and representative ^31^P MRS spectra from sham, vehicle- and albiglutide-treated hearts are presented with the prominent high-energy phosphate peaks visible (i.e., γ-ATP at −2.4 ppm, α-ATP at −7.5 ppm, β-ATP at −16 ppm, PCr at 0 ppm, intracellular inorganic phosphate (P_i_(in)) at 4.9 ppm, and extracellular inorganic phosphate (P_i_(ex)) at 5.1 ppm) (C). The resulting PCr/ATP ratio (D) PCr/Pi ratio (E) and cellular pH (F) are shown. Absolute whole heart ATP and PCr concentrations are shown in (G) and (H). Values are presented as mean ± SEM. *p<0.001, ^‡^p<0.01 and ^§^p<0.05 vs. sham; ^†^p<0.01 and ^II^p<0.05 vs. vehicle.

Cardiac MRI was used to assess LV function at 24 h post-I/R injury. Although the vehicle-treated rats had significantly reduced LV ejection fractions and increased LV end systolic volumes compared to sham, albiglutide treatment prevented the deleterious cardiac remodeling and function decline (Figure 6A, B). ^31^P MRS spectra from sham, vehicle-and albiglutide-treated hearts are presented in Figure 6C with the prominent high-energy phosphate peaks labeled (i.e., γ-ATP at −2.4 ppm, α-ATP at −7.5 ppm, βATP at −16 ppm, PCr at 0 ppm, and P_i_ at 4.9–5.1 ppm). As a result of I/R injury, the PCr peak is visibly decreased and P_i_ peak increased at the expense of maintaining steady state ATP concentrations via the creatine kinase enzyme reaction. The PCr/ATP ratio was variable and while it appeared to be lower in the vehicle group, the trend was not significant (Figure 6D). However, whereas the PCr/P_i_ ratio and the cellular pH were decreased in the vehicle group vs. sham (p<0.05), albiglutide prevented a change in these parameters (Figure 6E, F, respectively). Additionally, absolute whole heart ATP and PCr concentrations were markedly lower in vehicle-treated hearts compared to sham and albiglutide-treated hearts. (Figure 6G, H).

## Discussion

In the present study, it was demonstrated for the first time that albiglutide reduces myocardial infarct size in the experimental setting of acute myocardial I/R injury. The cardioprotective effect of albiglutide was further confirmed by the enhancement of post-ischemic cardiac function and energetic profile. In addition, assessment of [^3^H]-2-deoxyglucose uptake *in vivo* revealed that albiglutide markedly increased cardiac glucose uptake. Furthermore, in the Langendorff perfused heart, albiglutide exhibited an insulinomimetic effect by directly increasing cardiac glucose uptake and decreasing lactate efflux. The decreased lactate efflux was also associated with a direct increase in lactate oxidation which ultimately reflected a shift in cardiac metabolic substrate utilization with albiglutide that increased the relative carbohydrate oxidation versus fat oxidation. These results indicate that the infarct size-limiting effect of albiglutide was associated with an increase in glucose disposal, an increase in aerobic glycolysis, and an increase in lactate oxidation which resulted in an improved cardiac energetic state. These findings suggest that in addition to providing peripheral glycemic control, albiglutide may have direct therapeutic potential for improving cardiac function in the setting of myocardial ischemic injury.

Albiglutide exhibited acute pharmacological effects consistent with the systemic activation of the GLP-1 receptor [29]. These effects include body weight loss, reduced food intake, increased insulin secretion and reduced blood glucose following treatment. Treatment in the present study was initiated two days prior to myocardial ischemia as it was determined that albumin was maximally equilibrated with the heart after 48 hrs and consequently cardiac albiglutide exposure is greatest at this time (data not shown). Therefore, it is not known whether cardioprotection could be rendered if therapeutic dosing was initiated post-ischemia. Further investigation is necessary in order to address the potential for this therapeutic paradigm with albiglutide. In addition, the cardioprotection was observed with plasma concentrations as low as 17 nM which is within the pharmacological dosing range in type 2 diabetic patients in the clinic [16].

Myocardial infarct size is the gold standard for assessing myocardial I/R injury. Numerous studies examining the cardioprotective benefit of GLP-1 [4]–[7], exentatide [7], [9] and liraglutide [10] in models of I/R have been performed. As in the case of our study the cardioprotection observed with these GLP-1 agents was following pre-treatment with drug [4]–[7], [9], [10] while GLP-1 in the presence of a DDP-IV inhibitor has been shown to be beneficial in therapeutic mode (i.e. administration of drug post-infarct) [4]. Additionally, a few large animal cardioprotection studies using GLP-1 and mimetics in dog [13] and pig [9], [11] have been performed. While the evidence is clear that treatment with GLP-1 or mimetic prior to ischemic insult renders cardioprotection in rodents, there have been conflicting results in these larger animal studies. The study by Kristensen et al. [11] showed that pre-ischemic administration of liraglutide for 3 days prior to ischemic insult did not result in cardioprotection in the pig. Importantly, infarct size was measured 2.5 h after reperfusion in that study, whereas infarct size was determined 24 h post-reperfusion in the present study. Previous studies indicate that reperfusion time affects myocardial infarct size measurements [30], [31]. It is possible that the different experimental protocol such as reperfusion time could account for the negative outcome observed in the liraglutide study. In the current study, the area at risk was comparable between vehicle- and albiglutide-treated rats suggesting that all the animals were subjected to a similar ischemic insult. In addition, recombinant human albumin alone at a dose of 2.7 mg/kg was administered to the rats as a control experiment without having any effect on myocardial infarct size (data not shown), further confirming the infarct size-limiting effect of albiglutide.

The cardioprotective effect of albiglutide was further confirmed by improvements in cardiac function post-ischemia. The elevation of left ventricular end-diastolic pressure and reduction in left ventricular contractility, as assessed by dP/dt_max_, induced by ischemia/reperfusion injury were blunted with albiglutide. Additionally, LV ejection fraction and ESV were normalized. The improvement in post-ischemic cardiac function by albiglutide may be via a direct inotropic effect on the heart with activation of GLP-1 receptor in the setting of cardiac dysfunction [7], [14], [32], or via an indirect effect secondary to its reduction in myocardial infarct size. However, albiglutide exhibited no direct inotropic effect in control animals (data not shown). Indeed, reports showing the direct insulinomimetic effects of GLP-1 in both rodent perfused hearts [6], [7], [32], as well as in chronically instrumented dogs [13], [14], argue that an increase in glucose metabolism via direct insulinomimetic actions or via an indirect insulinotropic mechanism may in large part promote the cardioprotection afforded by GLP-1 receptor agonists. While albiglutide did reflect increased glucose uptake in perfused hearts and in insulin clamped hearts, in vivo, suggesting an insulinomimetic effect, one cannot rule out the possibility that the heart was predisposed to insulin sensitization upon two days of albiglutide treatment prior to the glucose disposal measurement.

GLP-1 was previously shown to increase cardiac lactate efflux concomitant with increased glucose disposal [6]. Albiglutide treatment resulted in a similar systemic elevation of plasma lactate at the high doses, although within the physiological range. This effect may be a result of the increased glucose disposal and glycolytic production of lactate by peripheral insulin sensitive tissue as plasma glucose was significantly reduced with albiglutide. Surprisingly, local lactate efflux in the heart was reduced following albiglutide treatment. Additionally, cardiac tissue lactate concentration was reduced with albiglutide along with the reduced lactate efflux and increased lactate oxidation. The consequence of this finding may be that there was decreased anaerobic glycolysis under conditions of increased glucose utilization with albiglutide. Perhaps tighter aerobic coupling of glucose oxidation was present. The increase in lactate oxidation observed in the albiglutide treated isolated hearts also confirms that the aerobic coupling of carbohydrate substrate oxidation was active. The heart, under normal conditions, oxidizes lactate [33]; however, following ischemic stress, the heart becomes a net producer of lactate [6] with intracellular lactate concentrations increasing significantly. This may be deleterious in a diabetic population where lactate acidosis is a significant liability not only peripherally, but also directly in the heart [34]. Our study indicates that albiglutide may be able to resolve these deleterious effects, but future cardioprotection studies in diabetic animal models are warranted.

Interestingly, although albiglutide promotes cardiac glucose uptake, the cardiac glycogen concentration was not increased in albiglutide-treated hearts, consistent with the notion that albigutide did not promote glycogenesis but increased cardiac glycolysis and glucose oxidation. Analysis of intermediary substrate handling as assessed by using a 1-^13^C glucose clamp allowed differentiation between carbohydrate oxidation (e.g. glucose, lactate, and pyruvate) and FFA oxidation by examining the degree of 4-^13^C glutamate enrichment dilution from unlabeled FFA [24]–[26]. There was no difference in alanine and lactate enrichment between vehicle- and albiglutide-treated hearts, but glutamate enrichment was significantly greater in albiglutide-treated hearts, reflecting that albiglutide increased carbohydrate versus fat oxidation in the heart and providing further evidence for the beneficial effect of increasing oxidative glucose handling in these hearts. A switch in myocardial substrate utilization from FFA towards glucose would provide an oxygen-sparing effect of approximately 12% for the same amount of ATP production [35]. This change in substrate utilization could provide the heart protective benefits in the setting of myocardial ischemic injury or possibly in the setting of the chronic dilated cardiomyopathy where impaired cardiac glucose metabolism and a reduced energetic state have also been observed [36], [37]. However, it is possible that other potential cardioprotective mechanisms such as anti-apoptosis via AKT signaling, and/or activation of RISK pathways may be contributing to the observed cardioprotection [4], [9]. Indeed, albiglutide significantly upregulated genes associated with cell survival such as IGF-1R, HIF-1α and AKT.

This more favorable energetic profile was substantiated by examining the high energy phosphate profile in the heart following I/R insult. There was a significant increase in the PCr/P_i_ ratio and ATP concentration in the area at risk in hearts treated with albiglutide. In addition, the reduction in pH following I/R injury was abrogated with albiglutide reflecting reduced anaerobic glycolysis which could lead to improved contractility [38]. The lack of benefit observed in the PCr/ATP ratio was most likely due to the decreased ATP concentration in the ischemic area of the vehicle group. The metabolic profile that was exhibited in albiglutide-treated hearts was also consistent with the altered gene expression profile observed in normal heart or non-ischemic heart following I/R injury as reflected by principal component analysis. This finding is a possible reflection of the ability of albiglutide to affect the metabolic profile and efficiency which ultimately translates to increased contractile efficiency in the adjacent border area to the ischemic tissue. The almost complete normalization of cardiac energetic and function in the heart while only reducing the infarct size by ∼26% suggests that there was a significant energetic and contractile benefit of albiglutide in the non-ischemic LV.

In summary, albiglutide, a novel GLP-1 analog with a long half-life, reduced myocardial infarct size and improved post-ischemic cardiac function and energetics following myocardial I/R injury. The observed benefits were associated with enhanced myocardial glucose uptake and shift toward a more energetically favorable substrate metabolism profile by increasing both glucose and lactate oxidation. These findings suggest that in addition to providing peripheral glycemic control, albiglutide may have direct therapeutic potential for improving cardiac energetic and function in the setting of myocardial ischemic injury.
